# Knowledge deficit on health promotion activities during pregnancy: the case for adolescent pregnant women at Chiladzulu District, Malawi

**DOI:** 10.1186/s12884-020-03386-w

**Published:** 2020-11-16

**Authors:** Lucy Ida Kululanga, Alice Kadango, Gaily Lungu, Diana Jere, Matthews Ngwale, Lily Caroline Kumbani

**Affiliations:** University of Malawi, Kamuzu College of Nursing, P.O. Box 415, Blantyre Campus, Blantyre, Malawi

**Keywords:** Adolescent pregnancy, Health promotion, Knowledge deficit

## Abstract

**Background:**

Adolescent pregnancy is a public health concern in Malawi as it is associated with high risks of adverse pregnancy outcomes. Almost 29% of adolescent women aged 15–19 years are already mothers and adolescent fertility rate is also high estimated at 136 per 1000 women. Therefore, the aim of the study was to explore knowledge of pregnant adolescents on importance of antenatal care and health promotion during pregnancy.

**Methods:**

A qualitative descriptive design was used to solicit information on significance of antenatal care and how adolescents promote their health during pregnancy. Data was collected from 77 pregnant adolescents, purposively sampled from Namitambo and Namadzi Heath Centres in Chiladzulu District, Malawi. A semi-structured interview guide was used for data collection. Data were analysed manually following principles of qualitative content analysis.

**Results:**

Themes that emerged from the qualitative data included: knowledge deficit on the purpose and benefits of antenatal care; knowledge deficit on services offered at antenatal care clinic; knowledge deficit on danger signs during antenatal period and antenatal emergency care; knowledge deficit on effects of alcohol and smoking; knowledge deficit on nutrition during pregnancy; and knowledge deficit on importance of rest during pregnancy.

**Conclusion:**

This study has shown knowledge deficit among adolescent mothers that may contribute to poor pregnancy outcomes. Several factors could be attributed to such knowledge deficit. Therefore, healthcare systems and healthcare professionals have a responsibility to enhance health literacy of pregnant adolescents with an ultimate goal of improving maternal and neonatal health outcomes.

## Background

Adolescents are persons aged 10 to 19 years [1] and adolescent pregnancy refers to a birth rate per 1000 teenage women [2]. Adolescent pregnancy is one of the major reproductive health challenges in the world. For instance, 21 million adolescent girls become pregnant and 12 million give birth every year and the majority of these births happen in low-and middle-income countries [3]. According to a World Health Organization report, global adolescent birth rate was 44 per 1000 girls aged 15–19 years old [4]. African countries are leading in adolescent pregnancy; for instance Niger is topping the list with 203.604 births per 1000 teen women, followed by Mali (175.4438), then Angola (166.6028), Mozambique (142.5334), Guinea (141.6722), Chad (137.173), Malawi (136.972) and Cote d’Ivoire (135.464, 2].

In Malawi, 29% of adolescent women aged 15 to 19 years are already mothers or pregnant with their first child and adolescent fertility rate is higher in rural areas estimated at 31% than in urban areas which is estimated at 21% [5]. Some of the factors associated with adolescent pregnancy are early marriages, poor social and economic support, curiosity and peer pressure, lack of comprehensive sexuality education, poor reproductive health services provision and poor attitudes of health workers in the provision of contraceptives to adolescents [3].

Adolescent pregnancy has been associated with higher risks of adverse pregnancy outcomes to both the mother and the baby. These poor outcomes to the mother include, eclampsia, anaemia, puerperal endometritis and systemic infection [3] while to the baby the outcomes are low birthweight, preterm delivery, severe neonatal conditions and neonatal mortality [6, 7]. In addition, adolescent mothers are more likely to develop mental health disorders such as depression compared to nonpregnant adolescents [8, 9]. Furthermore, adolescent pregnancy is a risk factor for malnutrition, low mental and physical development, inappropriate social connection with parents and poor education, social stigma and domestic violence [10, 11].

There is a great realization globally that adolescents are a special group that require responsive antenatal care [12]. Antenatal care is defined as the care provided by skilled healthcare professionals to pregnant women and adolescent girls to ensure optimum health conditions for both mother and baby during pregnancy [13]. Appropriate antenatal care is critical for health promotion of the mother resulting in good maternal and neonatal health thereby reducing maternal and neonatal mortality [14]. Antenatal care has been associated with positive pregnancy outcomes such as reduced probability of a woman giving birth to a low birth weight baby, reduced stunting and underweight in babies, reduced probability of neonatal and infant mortality [15]. The benefits of antenatal care could be attributed to a wide range of services that can prevent, detect and treat risk factors early on in the pregnancy [15]. However, studies on antenatal care services in Malawi have reported that women are dissatisfied with care [16, 17]. Reasons for dissatisfaction include poor quality of care, limited understanding on the importance of antenatal care, long waiting periods at the clinic and unsupportive clinic environment [17]. The impetus to conduct this study was the high adolescent pregnancy rate in Chiladzulu District estimated at 48% which is higher than the national rate of 29% [5]. However, through anecdotal observation, it was noted that there was no intervention to equip pregnant adolescents with knowledge, practices and attitudes on health promotion and risk identification during pregnancy. Therefore, this project aimed at developing an intervention which would promote knowledge, practices and attitudes on health promotion and health seeking behaviours among pregnant adolescents. The first phase of the project was to collect baseline data to determine the knowledge of pregnant adolescents on health promotion during pregnancy. The information that was obtained informed the modification of the antenatal education manual (Malawi Integrated Manual for Maternal and New-born Health 2015) to suit the needs of the pregnant adolescents in our project sites. This paper discusses the findings of the baseline assessment.

## Methods

### Aim of the study

The aim of the study was to explore knowledge of pregnant adolescents on importance of antenatal care and health promotion during pregnancy.

### Study design

The study used qualitative descriptive design to explore knowledge on health promotion activities during pregnancy among pregnant adolescents in a rural setting in Malawi. Qualitative descriptive design is a method of choice when a description of a phenomenon is desired [18] and information is sought for intervention development or refinement, conceptual clarification underlying scale development and needs assessments, especially in vulnerable populations [19]. Doody and Bailey stated that the goal of qualitative descriptive design is to provide a rich description of the experience and ‘*not “discovery” as is the case in grounded theory, “explain” or “seeking to understand” as with ethnography, “explore a process” as is a case study or “describe the experiences” as is expected in phenomenology’* [20]*.* One of the characteristics of qualitative descriptive design is flexibility in commitment to a theory or framework when designing and conducting a study in that the researchers may or may not decide to begin with a theory of the targeted phenomenon and do not need to stay committed to a theory or framework if their investigations take them down another path [21]. In view of the foregoing, qualitative descriptive design was used because of the aim of the study which was to assess knowledge of pregnant adolescents on importance of antenatal care and health promotion during pregnancy. Thus, the study design enabled the researchers to solicit straight descriptions of the knowledge that the pregnant adolescents had.

### Setting of the study

The study was conducted at Namitambo and Namadzi Health Centres in Chiradzulu District which were purposively selected. This district has the highest rate of adolescent pregnancy estimated at 48% compared to the estimated national rate of 29% in 2015–2016 in Malawi [5]. Both sites provide antenatal care to all pregnant women who come for care on a particular day as is the case in all health facilities. There are no specific services that target the needs of pregnant teenagers.

### Sample size

Seventy-seven adolescent women were interviewed, 37 from Namitambo health centre and 40 from Namadzi health centre**.** The sample size was decided apriori [22] as well as determined based on informational needs, and the guiding principle of data saturation. Data saturation is sampling to the point at which no new information is obtained and redundancy is achieved. In this study, data saturation was reached at participant number 30 at Namitambo Health Centre and 32 at Namadzi Health Centre. However, we went beyond point of saturation to ensure that no new or major concepts emerge. Moser and Korstjens suggest that when designing a qualitative sampling plan for content analysis, 15–20 interviews could be a starting sample size but this can vary depending on data saturation [23]. The sample size was suitable because it was large enough to uncover a variety of opinions of the potentially detailed data that can be generated from each participant [24]. The participating adolescent women were purposively selected with focus on those who were pregnant for the first time and had at least attended antenatal care clinic once.

### Data collection

The research team which comprises expert midwives and community health nurses developed the interview guide which was based on the ‘Malawi Integrated Manual for Maternal and newborn care 2015’ (antenatal matrix which includes health promotion messages and activities that each woman and her family needs to have for their care during perinatal period). The interview guide was validated by a team of expert midwives from Kamuzu College of Nursing.

Prior to data collection, the interview guide was pretested at Mauwa Health Centre, in Chiladzulu District, for validity by one of the researchers and research assistants. Six pregnant adolescents participated in the pre-test which was conducted in 3 days. The interviews lasted about 25–30 min. Appropriate changes regarding clarity of questions were made.

The participants were identified by the health personnel at the respective health facilities, and were referred to the study team. Numbers were used to identify the participants and no names were used. In addition, the research team were not part of the health care workers at the study settings. This enhanced objectivity during interviews. Participants were given information about the study and then asked if they were willing to participate in the study.

Data were collected from pregnant adolescents through in-depth interviews using a pre-tested semi-structured interview guide. The interview guide had 17 questions (see appendix 1). Information was collected on importance of antenatal care and knowledge pertaining to issues related to health promotion during pregnancy. Each participant was interviewed once by researchers and research assistants who were trained by the researchers on how to use the data collection tools and obtain informed consent. The in-depth interviews lasted 10 to 45 min with an average of 22 min. Data collectors summarized the key findings after each session and presented them to the participant as a measure of data validation [25]. Data collection was stopped when data saturation was achieved, thus when participants did not add any new information to the already collected data [26]. Data credibility was achieved through collection of data at two different sites, Namitambo and Namadzi Health Centres. In addition, participants’ responses we verified by repeating what had been discussed to validate the data.

### Data analysis

Data were analysed manually following qualitative content analysis methods [27] and systematic emergent coding principles [28]. The codes used to categorize data emerged from the data following the following steps: step 1: developing codes by using short phrases to represent an idea and all codes were assigned meaningful codes. Step 2: identifying themes, patterns and relationships.

Step 3: summarizing the data. At this last stage we linked research findings to research objectives. Thus, the themes were inductively developed from the data. Prior to analysis, the audio recorded data were transcribed verbatim to capture the whole interview and discussion. Each transcribed interview was translated into English as the interviews were conducted in Chichewa (vernacular language in Malawi). The transcripts were read several times to ensure full understanding of the meanings that were coming out from the data. Firstly, four research team members independently reviewed one script and came up with a set of codes that formed a checklist. Secondly, the research team members compared notes and reconciled any differences that showed up on the initial checklists. Thirdly, the research team consolidated the checklists to independently apply coding. Fourthly, the research team checked the reliability of the coding using inter-coder reliability using *Kappa (*k > 0.75 is excellent agreement; k 0.4–0.75 is intermediate to good agreement; k < 0.4 is poor) [29]. The inter-coder agreement in this study was above 0.75 which was acceptable. Then coding was applied to the whole data set. Codes were examined and similar codes were categorized and organized into themes. The themes were refined by checking them against the data to ensure that they correctly represent the data. Then the components of each theme were defined and explained.

## Results

### Demographic characteristics of the participants

The participants’ age ranged between 14 to 19 years with an average of 18.13 years and a modal value of 18 years. All participants had some form of formal education where 58.4% (*n* = 45) had primary education while 41.6% (*n* = 32) had secondary education. Almost 70 % (*n* = 54) of the participants were married, 20.8% (*n* = 16) had never been married, 5.1%(*n =* 4) were divorced, 1.2% (*n =* 1) was a widow and 2.6% (*n* = 2) participants’ marital status was not indicated. Almost eighty -4 % (*n* = 65) of the participants were prim-gravidas and 9% (*n* = 7) were multi-gravidas. All the participants indicated that they were engaged in some sort of income generating activity. However, only 25.9% (*n* = 20) indicated to have adequate food throughout the year while 62.3% (*n* = 48) indicated to have inadequate food and 11.7% (*n* = 9) indicated to be struggling to find food.

### Participants’ knowledge on issues related to pregnancy and antenatal care

Themes that emerged from the qualitative data namely: knowledge deficit on the purpose and benefits of antenatal care; knowledge deficit on services offered at antenatal care clinic; knowledge deficit on danger signs during antenatal period and antenatal emergency care; knowledge deficit on effects of alcohol and smoking; knowledge deficit on nutrition during pregnancy; and knowledge deficit on importance of rest during pregnancy.

### Knowledge deficit on the purpose and benefits of antenatal care

Participants were asked about the purpose and benefits of antenatal care. Most of their responses denoted that they lacked insight about purpose and benefits of antenatal care. Most of the responses portrayed antenatal clinics as a place to receive bed-nets, health passport books, and to have an HIV test. The participants’ responses did not show the relationship between the services they received at the clinic and the wellbeing of the mother and the unborn baby. Some participants said:

*‘I received a net and a health passport’.... ‘The net is important for malarial prevention’. (ID 149, Namitambo).*

*‘The mother knows the status of her body to continue taking care of her body’ (ID 149, Namitambo).*

### Knowledge deficit on services offered at antenatal clinic

When the participants were asked about the services offered at antenatal clinic and their significance to the mother and foetus, some participants mentioned having their weight and blood pressure checked, HIV testing, receiving iron tablets, Tetanus Toxoid vaccine, and bed nets and counselling sessions. The significance of the services to the mother included: weight is checked for adjustment of intake of food; blood is examined for malaria and levels because the mother has to have enough blood. In addition, HIV is also tested for the mother to start treatment and for the baby to be protected from HIV. *Other participants expressed:*

*‘The services that we get at the antenatal clinic include: blood pressure check, TTV, we receive medicines and bed nets, have an HIV test, and the nurses listen to the baby’s heartbeat.’ (ID 151, Namitambo).*

*‘The importance of antenatal care is that weight is checked so that you can have the required adjustment of intake of food; blood levels are checked to ensure that the body should have enough blood; and bed nets are given to prevent mothers from suffering from malaria and skin rashes; If you follow the counselling you can be aware of other things like the date of delivery’... in addition, ‘The baby becomes healthy if the body weight is increasing but if it is decreasing the baby cannot be healthy; enough blood helps the baby to be strong and will deliver a healthy baby’. (ID 55, Namitambo).*

‘The new born baby should not be infected by HIV’ (ID 149, Namitambo).

### Knowledge deficit on danger signs during antenatal period and antenatal emergency care

The participants were asked to mention danger signs during antenatal period and antenatal emergency care. Most of the participants demonstrated lack of knowledge on danger signs and emergency care during antenatal period. Few participants mentioned the following as danger signs during antenatal period: rupture of membrane, back pains, headache, lower abdominal pains, swollen legs and haemorrhage as indicated below:

*‘Some of the danger signs are haemorrhage and headache and lower abdominal pains.’ (ID 151, Namitambo).*

*‘Signs of danger are swollen legs, severe headache, haemorrhage and stomach pains.’ (ID 152, Namitambo).*

### Knowledge deficit on effects of alcohol and smoking

The participants mentioned several effects of alcohol and smoking to both the mother and the baby. Some of the effects of alcohol consumption to the mother according to the participants are that it causes mental illness, destroys intestines, weakens the mother’s body, destroys the lungs and hastens development of Tuberculosis. Some of the effects of alcohol to the baby include: foetal death, stillbirth, the baby can be born with malnutrition, the baby will be a drunkard, the baby will become mentally disturbed and the baby can develop Tuberculosis.

The participants mentioned the effects of smoking to the mother and baby that it disturbs the brain, destroys the lungs and the baby becomes mentally disturbed. In addition, the baby can be born with malnutrition.

*“The mother becomes mentally disturbed; she cannot eat properly. The baby can become malnourished.” (ID 152, Namitambo).*

*‘Smoking disturbs the brain of the mother and destroys the lungs of the baby.’ (Namitambo, ID 158).*

### Knowledge deficit on the importance of good nutrition during pregnancy

When the participants were asked on the importance of good nutrition during pregnancy, majority said that good nutrition makes the body strong and the baby is born strong as narrated by some participants:

“The baby will be born with malnutrition or mental problem” (ID 149, Namitambo).

“Mother will have good health and the baby will be strong and healthy because food gives us good health and the baby is born healthy” (ID 151, Namitambo).

### Knowledge deficit on importance of rest during pregnancy

The participants presented several reasons regarding importance of rest during pregnancy which included prevention of dizziness, premature labour, precipitated labour, stomach pains and miscarriage. In addition, some participants mentioned that rest makes the body strong and prevents sickness. Some of them said:

“Lack of rest may lead to stomach pains and dizzy spells” (IDI 1 Namadzi).

“Overworking may precipitate labour”.

“I can have stomach pains because of not resting” (ID 150 Namitambo).

‘Rest is important for an expectant woman to prevent miscarriage’ (IDI 07, Namadzi).

“The body becomes strong, no sickness” (ID 155 Namitambo).

## Discussion

The findings of this study have shown that pregnant adolescent mothers in Chiladzulu District specifically from Namitambo and Namadzi health centre catchment areas have knowledge deficit in matters related to health promotion during pregnancy. The knowledge deficit could be linked to health literacy which is defined as the ‘degree to which individuals can obtain, process, and understand basic health information and services needed to make informed health decisions’ [30]. In Malawi, health education about pregnancy and childbirth is usually provided by health workers, mostly at a health facility. However, most of the participants in this study had been to the clinic only once and may not have been given all the necessary antenatal information as most of them, 84% (*n* = 65) were primigravida who had never been exposed to antenatal education before unlike those with subsequent pregnancies. Some of the factors that could influence knowledge deficit among pregnant adolescents may include lack of awareness about health benefits of ANC, late recognition of pregnancy, poverty, being single and lack of support system throughout pregnancy, long distance and difficult roads to the clinic, and indulgence in agricultural work [31]. Furthermore, adolescent mothers may have increased responsibilities and face shame and embarrassment that would need someone to support them psychologically [32].

One of the strategies that can assist pregnant adolescent mothers to cope during prenatal period is attending antenatal care which could assist them to be empowered with information that would improve their wellbeing during pregnancy [33]. Additionally, pregnant adolescents need innovative models of antenatal care [12] such as adolescent group antenatal care. Kearney, Kynn, Kraswell and Reed, indicated that adolescent mothers would gain high maternal satisfaction, peer support and value shared experience if they attend group antenatal care [34]. Antenatal care offers an opportunity for mothers to explore and share their experiences that would increase their participation in sexual reproductive health issues [35]. Furthermore, adolescent pregnant mothers need community-based social support strategies that may assist them to cope with pregnancy and prevent pregnancy adverse issues [32]. Community-led improvement initiatives play an important role in connecting pregnant women with services where they can be tested and treated to promote optimal health and prevent HIV transmission 36]. Community-led initiatives assist in many ways such as spreading messages about the importance of antenatal care, identifying pregnant women, linking pregnant women to the facility, and following them up [36]. It has been asserted that the Community-Based Health Planning and Services (CHPS) increased utilization and improved access to antenatal care; pregnant women benefitted from better technical process quality of ANC services under the CHPS initiative than that found in non-CHPS areas in Ghana [37, 38].

Health care system could be a contributing factor to the adolescents not attending ANC services. For instance, adolescent mothers do not like to be mixed with adult mothers due to lack of privacy in the health facilities and they also fear being abused by the adult mothers [39]. In most cases in Malawi, antenatal care does not specifically have services that would target the needs of pregnant teenagers as indicated in an exploratory study on pregnant adolescents’ perceptions of the antenatal care received at a health centre in Blantyre, Malawi which found that the care was inadequate, as it did not meet the adolescents’ expected standards and needs [12]. Similarly, a study done in India highlighted that there were persistent unmet quality standards like provision of equipment and supplies to address the needs of adolescent mothers [40]. On the other hand, services that are more adolescent friendly resulted in increased knowledge about reproductive health services to young people [40]. Banke- Thomas et al., further added that there is poor utilization of maternal health services by adolescents compared to older mothers and concluded that education and male involvement in maternal health care would improve utilisation of the services [41]. The assumption is that involvement of men in maternal services would increase use of the services by adolescent mothers through empowering them with enough financial resources to access the critical care [41]. The supposition is that antenatal attendance would enhance pregnant adolescents’ health literacy so that they can meaningfully participate in their health care and provide feedback on health services and decisions regarding their own care [1] which ultimately would improve maternal and neonatal health outcomes. Thus, health care providers have a duty to empower adolescent mothers with health information.

The study had some limitations such as use of nurses and midwives as research assistants who could have their own biases. However, professions and identities of the research assistants were concealed and their duty district was not Chiladzulu District. The study was limited to Chiladzulu District and only focused on pregnant adolescents who used the health facilities, as such the results may be limited in application. Nonetheless, the study brings out important issues that may adversely contribute to pregnancy outcomes among adolescent mothers.

## Conclusions

The unique finding of this study is that adolescent pregnant women have knowledge deficit on matters that will enable them to stay healthy during pregnancy and to give birth to a healthy baby. This may be attributed to several factors such as low educational level, being young and poor and lack of exposure to health information. Improving health literacy of the pregnant adolescents is the responsibility of the healthcare systems, and healthcare professionals. It is critical for pregnant adolescents to develop health literacy so that they can be proactive regarding their health and be able to make more informed decisions which can ultimately improve maternal and neonatal health outcomes.

## Data Availability

The dataset used and analysed in this study are available from the corresponding author on reasonable request.

## Appendix I: Interview guide for pregnant adolescents (English version)

NO __________________________

HEALTH FACILITY ___________________________

VILLAGE _________________________

TRADITIONAL AUTHORITY _________________________

ENUMERATOR’S NAME __________________________

DATE __________________________

SUPERVISOR’S NAME ___________________________

DATE ___________________________

**Knowledge on issues related to health promotion during pregnancy**

1. What activities do you know that take place at an antenatal Clinic?
  Probe: *What information do you know about activities done to pregnant mothers at the antenatal clinic?*
2. Do you know anything about danger signs that occur during pregnancy?
  Probe: *What is the impact of danger signs to adolescent mothers?*
3. What information do you know regarding what a pregnant woman can do to manage any danger sign in pregnancy at home?
  Probe: *Are there other ways used to manage danger signs in pregnancy at home?*
4. What information have you received at the Antenatal Clinic to day?
5. What care have you received at the Antenatal Clinic to day?
6. What are the benefits of attending antenatal care?

- To you as a pregnant woman?
- To your unborn child?

7. Do you know why midwives collect your blood sample during antenatal care?
  *Yes* … …………… … .. *No* … …………………… … …
  Probe:
  a. What tests do they do with the blood samples midwives collect during antenatal care clinic?
  b. What is the importance of these blood tests?

- to your unborn child?
- to you as a pregnant woman?

8. Do you know why midwives check your blood pressure when you come to the antenatal clinic?
  Yes____________ No____________________.
  **Probe:** If yes, why do midwives check your blood pressure when you come to the antenatal clinic
9. Do you know why midwives check your body weight when you come to the antenatal clinic?
  Yes_____________ No ____________________.
  **Probe:** If yes, why do midwives check your body weight when you come to the antenatal clinic
10. What have you learnt today during antenatal health education session?

Probe: *Explain the importance of these lessons during your perinatal period?*

11. What kind of food do you like to eat during pregnancy?
12. What are the benefits of good food to a pregnant woman?
13. What are the benefits of good food to the unborn child?
14. Which local herbs do you usually use during pregnancy?

Probe: *Can you name the herb, its indication and use?*

15. Do you take alcohol? Yes___________ No ___________
  **Probe:**
  a. Do you think alcohol is dangerous to a pregnant mother? Yes ______ No __.

b. If yes to question 15, why is alcohol dangerous to:

- a pregnant mother?
- Unborn child?

16. Do you smoke? Yes ___________ No _________________

**Probe:**

- Do you think smoking is dangerous to a pregnant mother? Yes _____ No ____
- If yes to question 16, why is smoking dangerous to:

- a pregnant mother.
- Unborn child

17. As a pregnant woman, do you have time to rest from your daily chores?
  Yes _______ No _______.

***Probe***: If yes*,* why is rest important to a pregnant mother?

Probe: If no, what are the implications of not having enough rest during pregnancy?

***Thank you so much for your participation. Have a safe journey back home.***

## Abbreviations

ANC: Antenatal care
BMI: Body mass index
COMREC: College of Medicine Research and Ethics Committee
HIV: Human immunodeficiency virus
TTV: Tetanus toxoid vaccine
SRH: Sexual and Reproductive Health
